# Engineering of Serine-Deamination pathway, Entner-Doudoroff pathway and pyruvate dehydrogenase complex to improve poly(3-hydroxybutyrate) production in *Escherichia coli*

**DOI:** 10.1186/s12934-014-0172-6

**Published:** 2014-12-16

**Authors:** Yan Zhang, Zhenquan Lin, Qiaojie Liu, Yifan Li, Zhiwen Wang, Hongwu Ma, Tao Chen, Xueming Zhao

**Affiliations:** Key Laboratory of Systems Bioengineering (Ministry of Education), Tianjin University, Tianjin, 300072 China; SynBio Research Platform, Collaborative Innovation Center of Chemical Science and Engineering (Tianjin), School of Chemical Engineering and Technology, Tianjin University, Tianjin, 300072 China; Edinburg-Tianjin Joint Research Centre for Systems Biology and Synthetic Biology, Tianjin University, Tianjin, 300072 China; Key Laboratory of Systems Microbial Biotechnology, Tianjin Institute of Industrial Biotechnology, Chinese Academy of Sciences, Tianjin, 300308 China; Department of Biochemical Engineering, School of Chemical Engineering and Technology, Tianjin University, Tianjin, 300072 China

**Keywords:** *Escherichia coli*, poly(3-hydroxybutyrate), L-serine deaminate, Entner-Doudoroff pathway, Pyruvate dehydrogenase complex

## Abstract

**Background:**

Poly(3-hydroxybutyrate) (PHB), a biodegradable bio-plastic, is one of the most common homopolymer of polyhydroxyalkanoates (PHAs). PHB is synthesized by a variety of microorganisms as intracellular carbon and energy storage compounds in response to environmental stresses. Bio-based production of PHB from renewable feedstock is a promising and sustainable alternative to the petroleum-based chemical synthesis of plastics. In this study, a novel strategy was applied to improve the PHB biosynthesis from different carbon sources.

**Results:**

In this research, we have constructed *E. coli* strains to produce PHB by engineering the Serine-Deamination (SD) pathway, the Entner-Doudoroff (ED) pathway, and the pyruvate dehydrogenase (PDH) complex. Firstly, co-overexpression of *sdaA* (encodes L-serine deaminase), L-serine biosynthesis genes and *pgk* (encodes phosphoglycerate kinase) activated the SD Pathway, and the resulting strain SD02 (pBHR68), harboring the PHB biosynthesis genes from *Ralstonia eutropha*, produced 4.86 g/L PHB using glucose as the sole carbon source, representing a 2.34-fold increase compared to the reference strain. In addition, activating the ED pathway together with overexpressing the PDH complex further increased the PHB production to 5.54 g/L with content of 81.1% CDW. The intracellular acetyl-CoA concentration and the [NADPH]/[NADP^+^] ratio were enhanced after the modification of SD pathway, ED pathway and the PDH complex. Meanwhile, these engineering strains also had a significant increase in PHB concentration and content when xylose or glycerol was used as carbon source.

**Conclusions:**

Significant levels of PHB biosynthesis from different kinds of carbon sources can be achieved by engineering the Serine-Deamination pathway, Entner-Doudoroff pathway and pyruvate dehydrogenase complex in *E. coli* JM109 harboring the PHB biosynthesis genes from *Ralstonia eutropha*. This work demonstrates a novel strategy for improving PHB production in *E. coli*. The strategy reported here should be useful for the bio-based production of PHB from renewable resources.

## Background

Polyhydroxyalkanoates (PHAs) are diverse polyesters synthesized by a variety of microorganisms as intracellular carbon and energy storage compounds in response to environmental stresses [1]. Since PHAs possess thermoplastic or elastomeric properties and are completely biodegradable, PHA bioplastics offer an exciting alternative to petrochemical-derived plastics [2]. Poly(3-hydroxybutyrate) (PHB) is the most wide spread and best-characterized member of PHAs and many different fermentation strategies and recovery methods have been developed for its production as a model polymer [3].

**Figure 1:**
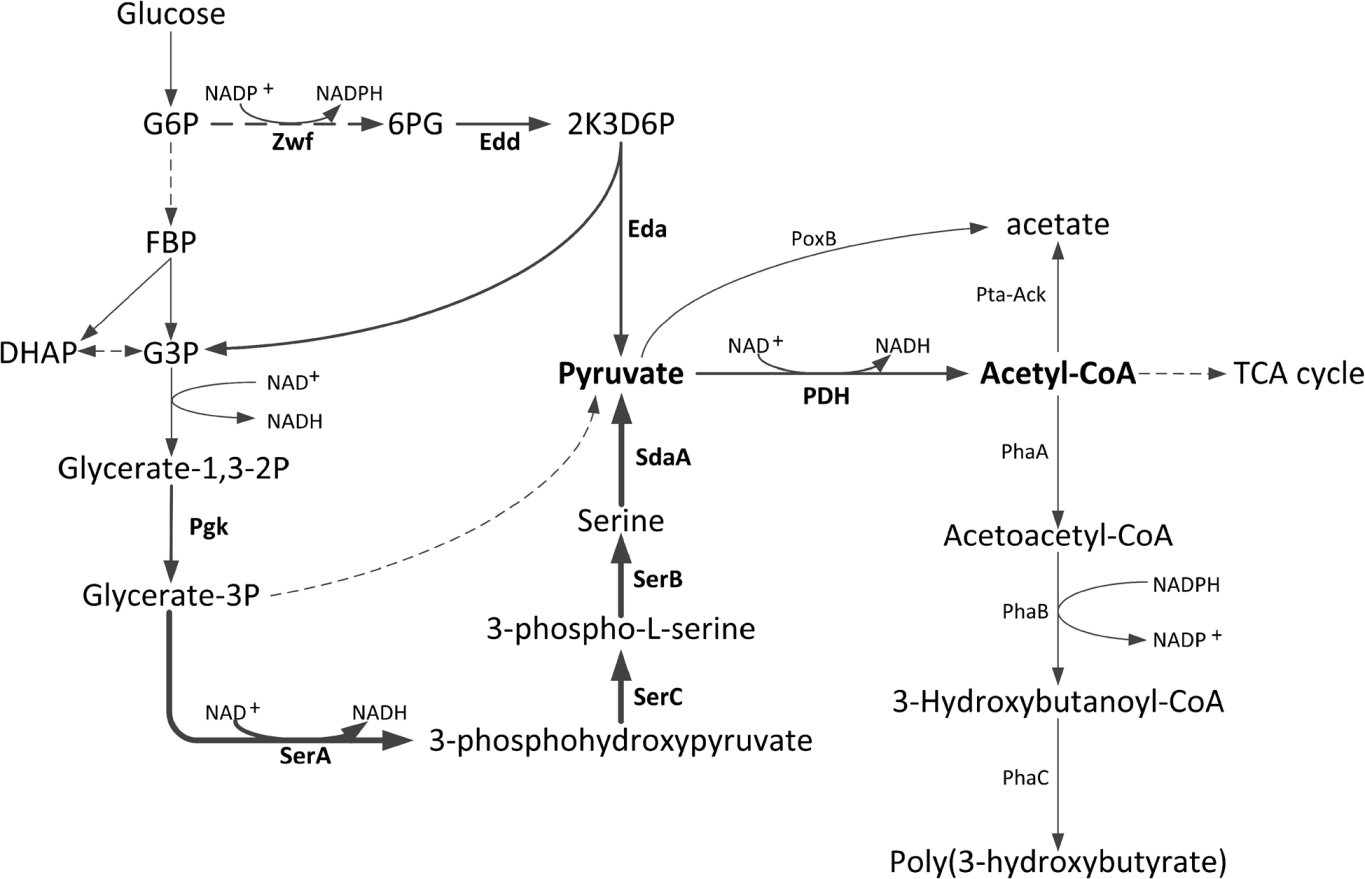
**Schematic representation of the SD and ED metabolic pathways in PHB accumulation recombinant** ***E.coli*** **.** Dashed lines indicate multiple enzymatic steps. The bold lines indicate the enzymes of SD pathway including reactions catalyzed by SerACB and SdaA. The enzymes that had been overexpressed in this work were shown in boldface. G6P, glucose-6-phosphate; FBP, fructose-1,6-bisphosphate; G3P, glycerahyde-3-phosphate; DHAP, dihydroxyacetone phosphate; 6PG, 6-phosphate-gluconate. Enzymes are as follows: Zwf, glucose 6-phosphate-1-dehydrogenase; Edd, phosphogluconate dehydratase; Eda, 2-keto-3-deoxygluconate 6-phosphate aldolase; Pgk, phosphoglycerate kinase; SerA, D-3-phosphoglycerate dehydrogenase; SerB, phosphoserine phosphatase; SerC, 3-phosphoserine aminotransferase; SdaA, L-serine deaminase I; PoxB, pyruvate oxidase; Pta, phosphate acetyltransferase; Ack, acetate kinase; PhaA, β-ketothiolase; PhaB, NADPH-dependent acetoacetyl-CoA reductase; PhaC, PHB synthase.

In the majority of native PHB-accumulating species, PHB is synthesized from acetyl-CoA by a sequence of three enzyme reactions catalyzed by β-ketothiolase, acetoacetyl-CoA reductase and PHB synthase, encoded by *phaA*, *phaB* and *phaC*, respectively (Figure 1). Recombinant *E. coli* harboring the exogenous PHB synthetic pathway was one of the most frequently used hosts for biopolymer production because of its advantages such as having a wide range of utilizable carbon sources, accumulating of large amounts of polymers with a high level of productivity, high cell density fermentation, and lacking PHA degradation system.

There has been a range of studies to evaluate PHB production in *E. coli*. Enhancing the availability of the precursor acetyl-CoA or/and cofactor NADPH increased the production of PHB. By overexpressing the fructose-bisphosphate aldolase (encoded by *fbaA*) or/and triosephosphate isomerase (encoded by *tpi*) [4], the recombinant *E. coli* accumulated more PHB than the reference strains due to the increase in acetyl-CoA concentration. By inactivating the phosphoglucose isomerase (encoded by *pgi*) gene, more NADPH was produced from pentose phosphate (PP) pathway, and eventually the PHB production was enhanced [5]. Transketolase (encoded by *tktA*) or transaldolase (encoded by *talA*) was used to improve the metabolism in non-oxidative PP pathway to enhance the production of PHB [6,7]. Overexpressing glucose-6-phosphate dehydrogenase (encoded by *zwf*) and 6-phosphogluconate dehydrogenase (encoded by *gnd*) in oxidative pentose phosphate pathway increased the PHB production in *E. coli* by increasing the NADPH availability [8]. In addition, to increase product yields, NADPH levels have been manipulated in the past by overexpressing the NADP^+^-dependent D-glyceraldehyde-3-phosphate dehydrogenase from *Streptococcus mutans* [9].

In *E. coli*, acetyl-CoA was derived mostly from pyruvate which is a key intermediate in catabolic and biosynthetic reactions. Most pyruvate was synthesized through the coupled mechanism of glucose transport by the phosphotransferase transport system or the glycolytic pathways including Embden-Meyerhof-Parnas (EMP), Entner-Doudoroff (ED), and PP pathway [10]. *E. coli* only produced two mole of NADH per mole of glucose through the EMP pathway, and caused the carbon loss through the PP pathway. Compared with these two pathways, ED pathway enables the strain to produce one molecular NADPH, a direct cofactor for the PHB production, without the carbon loss. Moreover, previous research based on proteome analysis revealed that ED pathway plays an important role during PHB production from glucose [11,12]. In *E. coli*, pyruvate also can be synthesized from L-serine by L-serine deaminase [13]. L-serine deaminases (encoded by *sdaA*, *sdaB* and *tdcG*) [14] catalyze the conversion of L-serine to pyruvate and ammonia, which may be employed to improve the PHB production in *E.coli*.

In this research, combined engineering of the Serine-Deamination Pathway (SD pathway, shown in Figure 1) with ED pathway resulted in enhanced PHB production, but accompanying with pyruvate accumulation. Overexpressing PDH complex eliminated the pyruvate accumulation and led to further improvement of the intracellular acetyl-CoA concentration and PHB production. Finally, the metabolically engineered *E. coli* strain was able to synthesize significant amount of PHB from different carbon sources such as glucose, xylose and glycerol.

## Results and discussion

### Overexpressing L-serine deaminase for improved PHB production

**Figure 2:**
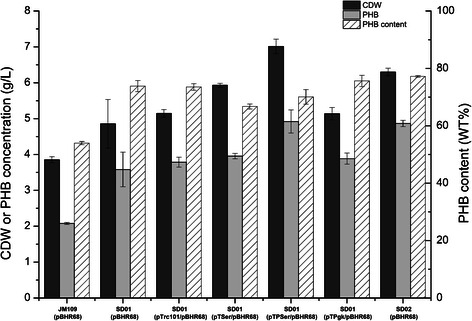
**Comparison of CDW, PHB concentration and PHB content in recombinant** ***E. coli*** **strains.** Histogram shows the mean of three biological replicates, and error bars show standard deviations.

Under aerobic or anaerobic conditions, acetyl-CoA was derived mostly from the decarboxylation of pyruvate which respectively catalyzed by the PDH complex or pyruvate-formate lyase in *E.coli* [15,16]. Pyruvate was formed mostly from several glycolysis pathways. Moreover, L-serine derived from D-3-phosphoglycerate can be catalyzed to pyruvate and ammonias by L-serine deaminase. However, L-serine also can be cleaved into glycine and one carbon unit by serine hydroxymethyltransferase (SHMT, encoded by *glyA*), and be used as a building block for protein synthesis. Considering the competitive pathways existing at L-serine node, we firstly overexpressed the L-serine deaminase to enhance the conversion of L-serine into pyruvate. In *E. coli*, L-serine is deaminated by three L-serine deaminases, which are encoded by *sdaA*, *sdaB* and *tdcG*, respectively. L-serine deaminases I (SdaA) which is encoded by *sdaA* gene, is responsible for L-serine degradation in minimal media [14]. Moreover, a previous report has shown that *sdaA*-overexpressing *Corynebacterium glutamicum* could grow in the medium using L-serine as the sole carbon source [17]. Thus, in order to activate the SD pathway, *sdaA* was selected to be overexpressed by replacing the native *sdaA* promoter with a strong constitutive promoter *trc* in JM109, resulting in strain SD01 (JM109, P_*trc*_*-sdaA*). To test the effect of overexpressing *sdaA* on PHB production, SD01 and JM109 were both transformed with the plasmid pBHR68 which consists of the PHB biosynthesis genes from *Ralstonia eutropha*, creating strains SD01 (pBHR68) and JM109 (pBHR68). The PHB production of SD01 (pBHR68) was 3.58 g/L, 1.72-fold of that of JM109 (pBHR68) (Figure 2), and the PHB content is 73.8% of the cell dry weight (CDW) (Figure 2). These results suggested that overexpression of *sdaA* obviously “pulled” more L-serine to pyruvate and led to the improvement of PHB production. Thus, SD01 was chosen as the host for subsequent modifications to improve PHB production.

### Influence of gene overexpression in L-serine biosynthetic pathway on PHB production

It is reasonable that efficient conversion of L-serine to pyruvate requires sufficient availability of L-serine. To enhance the biosynthesis of L-serine, we overexpressed the genes of *de novo* L-serine biosynthetic pathway. L-serine is synthesized from D-3-phosphoglycerate by three reactions catalyzed by D-3-phosphoglycerate dehydrogenase, D-3-phosphoserine aminotransferase and phosphoserine phosphatase, which are encoded by *serA*, *serC* and *serB,* respectively (Figure 1)*.* D-3-phosphoglycerate dehydrogenase is regulated by allosteric end-product inhibition. Moreover, a published report has showed that a truncated D-3-phosphoglycerate dehydrogenase (PGDH) *serA*^*Δ197*^ was no longer inhibited by L-serine in *C. glutamicum* [18]. As such, we combined *serA*^*Δ197*^ together with *serB* and *serC* into an artificial operon driven by the constitutive promoter *trc*, creating plasmid pTSer. Strain SD01 was transformed with plasmid pTSer for activating the Serine-Deamination (SD) pathway. After 48h cultivation, 3.96 g/L PHB was accumulated by SD01 (pBHR68/pTSer), which was only slightly higher than that of the reference strain SD01 (pBHR68/pTrc101) (Figure 2).

However, the strategy of overexpressing s*erABC* mainly led to improvement of cell growth, rather than specific PHB accumulation (Figure 2). This phenomenon might be due to the difference between the kinetic parameters of the two enzymes, SHMT and SdaA. The K_m_ value for L-serine of SdaA is higher than that of SHMT (2.67 vs 0.3 mM [19,20]). The overexpression of *serABC* improved the availability of L-serine, which contributed much more to the reaction catalyzed by SHMT than the reaction catalyzed by SdaA. SHMT is the main source of C1 carbon and glycine for cell growth in *E.coli*, so residual cell dry weight was increased under this condition [21,22].

On the other hand, the k_cat_ value of SdaA is much higher than that of SHMT (436 vs 5 s^−1^ [19,20]), and the corresponding k_cat_/K_m_ values are 163.3 and 16.7 mM^−1^ · s^−1^, respectively. This means only when the intracellular concentration of L-serine is further improved to some extent, the serine deamination reaction can obviously overwhelm the competing reaction catalyzed by SHMT. Previous researches have shown that *pgk* (encodes phosphoglycerate kinase) overexpression could divert carbon flux into the D-3-phosphoglycerate pool and further promote the biosynthesis of L-serine [23]. To push more flux into the SD pathway, *pgk* gene was ligated into pTSer, creating plasmid pTPSer. The PHB production of SD01 (pBHR68/pTPSer) reached 4.92 g/L, increasing by 30.2% compared to that of SD01 (pBHR68/pTrc101) harboring the empty vector (Figure 2). However, when *pgk* was overexpressed alone, the PHB production of SD01 (pBHR68/pTPgk) showed no significant change, compared with that of SD01 (pBHR68/pTrc101). These results suggested that activating the SD pathway by co-overexpressing the L-serine deaminase, enzymes in L-serine biosynthesis pathway and phosphoglycerate kinase had a significant effect on PHB production.

**Figure 3:**
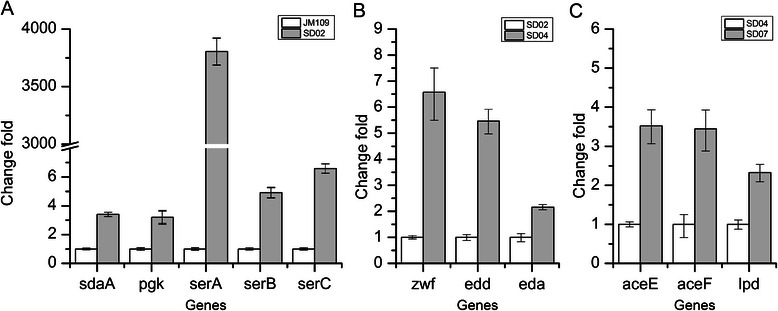
**Results of relative transcriptional level. A**. The transcription level of the genes in SD pathway; **B**. The transcription level of the genes in ED pathway; **C**. The transcription level of the genes in PDH. Histogram shows the mean of three biological replicates, and error bars show standard deviations.

Considering the instability of plasmid system and the metabolic burden resulted from plasmid replication [24,25], the *pgk*-*serABC* operon together with the fragment Trc-162 was integrated into the choromosome of SD01 at *serC* site, creating strain SD02 (SD01, P_Trc-162_-*SerABC*). The transcription levels of genes in SD pathway in strain SD02 were compared with that of the reference strain JM109 through RT-PCR analysis. As shown in Figure 3A, the genes of SD pathway have been successfully overexpressed. SD02 (pBHR68) accumulated 4.86 g/L PHB, which was almost the same as that of SD01 (pBHR68/pTPSer), but had a higher PHB content of 77.2% CDW (Figure 2). Therefore SD02 was selected for further engineering.

### Effect of combining gene overexpression in ED pathway with the SD pathway on PHB production

Redirecting the carbon metabolism into the ED pathway in the engineered strain is beneficial to PHB production, since it avoids the carbon loss and balance the NADPH need [12,26]. Moreover, enhancing ED pathway might increase the availability of pyruvate and further enrich the *in vivo* pool of acetyl-CoA. As such, we tried to test the effect of enhancing the ED pathway on PHB accumulation.

**Figure 4:**
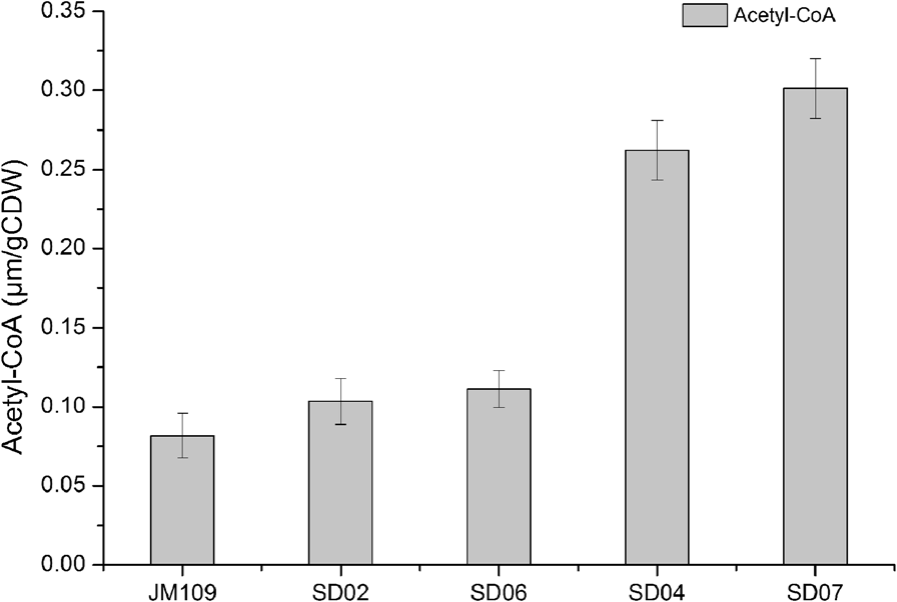
**Intracellular acetyl-CoA concentrations of recombinant** ***E. coli*** **.** The average cell dry weight for all of the strains was 0.38 g/liter per optical-density (OD_600_) unit of culture. Histogram shows the mean of three biological replicates, and error bars show standard deviations.

The ED pathway joins the oxidative pentose phosphate pathway to EMP pathway via two enzyme-catalyzed reactions. The two critical enzymes are phosphogluconate dehydratase and 2-keto-3-deoxygluconate 6-phosphate aldolase, which were encoded by *edd* and *eda,* respectively. In *E. coli*, the ED pathway has been shown to be inactive with glucose as the carbon source [27]. Thus, the upstream regulated region of the *edd*-*eda* operon in SD02 was replaced with a constitutive promoter J23119 (http://partsregistry.org/Part:BBa_J23119) for eliminating the repression of the ED pathway and activating the ED pathway, resulting in SD03 (SD02 P_*J23119*_*-edd*). Unexpectedly, strain SD03 (pBHR68) produced 2.90 g/L PHB which was a significant decrease compared with that of SD02 (pBHR68) (Table 1). Then fragment Trc-162 was inserted at the upstream of *zwf* gene in SD03 to further enhance the flux of ED pathway, creating strain SD04 (SD03, P_Trc-162_-*zwf*). The transcription levels of *zwf*, *edd* and *eda* genes in SD04 were up-regulated by 5.46, 4.57 and 2.16-fold, respectively (Figure 3B). PHB was accumulated to 3.39 g/L in SD04 (pBHR68), a 16.9% increase compared with that of SD03 (pBHR68), but was still lower than that of the parent strain SD02 (pBHR68) (Table 1). In addition, a small amount of pyruvate and acetate had been detected in the media of strains SD03 (pBHR68) and SD04 (pBHR68), but not detected in SD02 (pBHR68) (Table 1). This might be due to the improvement of glucose consumption after co-overexpressing the *edd, eda, zwf* genes (Table 1), which resulted in pyruvate accumulation in the medium. Moreover, the acetate was produced directly from pyruvate by pyruvate oxidase (encoded by *poxB*) in the JM109 derivative strains [28]. Thus it was possible that the accumulation of pyruvate and acetate retarded the cell growth and led to lower PHB production, although the intracellular acetyl-CoA concentration and the [NADPH]/[NADP^+^] ratio was increased (Figure 4, Table 2). So we tried to enhance the conversion of pyruvate to acetyl-CoA to eliminate the pyruvate accumulation and increase the PHB production.

**Table 1:** **Cell growth, PHB production, and by-products formation by strains study at the end of the cultivation**

**Strains** ^**a**^	**CDW (g/L)** ^**c**^	**PHB (g/L)** ^**c**^	**PHB content (%CDW)** ^**c**^	**Pyruvate (g/L)** ^**c**^	**Acetate (g/L)** ^**c**^	**Specific glucose uptake rate (g/g rCDW·h)** ^**b, c**^
JM109 (pBHR68)	3.85±0.09	2.08±0.03	54.0±0.57	ND	0.18±0.01	0.38±0.01
SD02 (pBHR68)	6.30±0.11	4.86±0.09	77.2±0.32	ND	ND	0.64±0.01
SD03 (pBHR68)	4.29±0.04	2.90±0.10	67.6±1.98	0.34±0.01	0.84±0.04	0.71±0.02
SD04 (pBHR68)	5.05±0.24	3.39±0.17	67.0±1.33	0.51±0.03	0.33±0.03	0.73±0.01
SD06 (pBHR68)	6.70±0.14	4.63±0.12	69.1±0.50	ND	ND	0.50±0.01
SD07 (pBHR68)	6.83±0.19	5.54±0.15	81.1±1.20	ND	ND	0.93±0.01

^a^strains were cultured for 48 h in mineral salt medium supplemented with 1 g/L yeast exact and 20 g/L glucose at 37°C at a rotation rate of 220 rpm under aerobic conditions;

^b^The specific glucose uptake rate was determined between 0 h and 24 h of the fermentation. rCDW: residual cell dry weight.

^c^Data were expressed as average values and standard deviations (SD) of three parallel studies.

**Table 2:** **Comparison of the intracellular NADP** ^**+**^ **, NADPH concentrations and [NADPH]/[NADP+] ratios of the recombinant strains without the PHB synthesis genes**

**Strains** ^**a**^	**NADP** ^**+**^ **(μmol/gCDW)** ^**b,c**^	**NADPH (μmol/gCDW)** ^**c**^	**[NADPH]/[NADP** ^**+**^ **] ratio** ^**c**^
JM109	0.99±0.02	0.72±0.02	0.73±0.02
SD02	1.00±0.02	0.73±0.01	0.73±0.01
SD04	0.91±0.04	0.84±0.03	0.92±0.01
SD07	0.86±0.02	0.77±0.03	0.90±0.01

^a^Cells were cultivated in MS medium containing 10 g/L of glucose at 37°C for 6 h.

^b^CDW The average cell dry weight for all of the strains was 0.38 g/liter per optical-density (OD_600_) unit of culture.

^c^The data shown are the average and standard deviations of three parallel experiments.

### Overexpression of the pyruvate dehydrogenase complex for enhanced PHB production

In *E. coli*, pyruvate is metabolized by the pyruvate dehydrogenase (PDH) complex and pyruvate oxidase during aerobic growth conditions [15,29]. To enhance the expression of PDH complex, fragment Trc-162 was inserted into the upstream of the PDH operon in SD02 and SD04 as mentioned in the methods section, resulting in strains SD06 (SD02, P_Trc-162_-*aceEF*) and SD07 (SD04, P_Trc-162_-*aceEF*). The transcript levels of genes encoding the PDH complex were presented in Figure 3C, which indicated that the genes of the PDH complex were successfully overexpressed. The PHB production of SD06 (pBHR68) was 4.63 g/L (69.1% CDW), no significant change compared with the reference strain SD02 (pBHR68) (Table 1), as well as the intracellular concentration of acetyl-CoA (Figure 4).

In contrast, the PHB concentration of the strain SD07 (pBHR68) was 5.54 g/L and PHB content was 81.1% CDW, significantly increased from 2.90 g/L of the parent strain SD04 (pBHR68). As expected, no trace of pyruvate and acetate was detected in the medium of SD07 (pBHR68) (Table 1), and the intracellular acetyl-CoA concentration of SD07 was further increased compared with that of SD04 (Figure 4). These indicated that the diversion of excess pyruvate to acetyl-CoA enhanced the availability of acetyl-CoA for PHB synthesis, and thus rebalanced the flux distribution at the pyruvate node.

### PHB fermentation using other unrelated carbon sources

Xylose is a major constituent of lignocellulose biomass, and glycerol is a major byproduct of petrochemical products, both of which have been utilized for the production of various PHAs as cheap carbon sources [30-32]. We also tested the PHB production performance of the recombinant strains by using xylose or glycerol as carbon sources. Compared to wild type strain, SD06 (pBHR68) and SD07 (pBHR68) showed significant increase in PHB production and PHB content (Table 1). However, no significant difference was observed between these two strains when xylose or glycerol was served as the sole carbon source (Table 3). The reason for this phenomenon was that xylose or glycerol turned into D-3-phosphoglyceraldehyde via a series of metabolic reactions and flow into the EMP pathway or SD pathway rather than the ED pathway under aerobic conditions. These results suggested that the strategy reported here should be useful for the bio-based production of PHB from different carbon sources.

**Table 3:** **PHB accumulation of the strains in xylose or glycerol**

**Carbon source**	**Strains** ^**a**^	**CDW (g/L)** ^**b**^	**PHB (g/L)** ^**b**^	**PHB content (%CDW)** ^**b**^
Xylose	JM109(pBHR68)	3.76±0.09	1.79±0.05	47.5±0.86
Xylose	SD06(pBHR68)	5.72±0.05	3.90±0.06	68.1±1.00
Xylose	SD07(pBHR68)	5.70±0.13	3.92±0.04	68.8±1.06
Glycerol	JM109(pBHR68)	3.38±0.08	1.37±0.06	38.4±1.23
Glycerol	SD06(pBHR68)	3.91±0.05	2.15±0.10	54.9±1.72
Glycerol	SD07(pBHR68)	3.79±0.07	2.12±0.08	56.0±1.03

^a^Bacteria were cultured for 48 h in mineral salt medium supplemented with 1 g/L yeast exact and 20 g/L xylose/glycerol at 37°C at a rotation rate of 220 rpm under aerobic conditions.

^b^Data were expressed as average values and standard deviations (SD) of three parallel studies.

## Conclusions

Through combined engineering of SD pathway, ED pathway and PDH, a recombinant *E. coli* strain was obtained which led to significantly enhanced PHB accumulation. The final strain produced 5.52 g/L PHB from glucose with a content of 81.11% CDW. A similar phenomenon was observed when xylose or glycerol was served as carbon sources. In conclusion, enhancing the availability of acetyl-CoA via engineering the SD pathway, ED pathway and the PDH complex offered an effective way for improving the PHB production in *E. coli* from different carbon sources.

## Methods

### Bacterial strains, primers, and plasmids construction

Bacterial strains and plasmids used in this study were listed in Table 4. *E. coli* DH5α was used for plasmid construction. The truncated *serA*^*Δ197*^ from *C. glutamicum* and *serB*, *serC* genes from *E. coli* with the synthesized ribosome binding sites (RBSs) were amplified by polymerase chain reaction (PCR) using the primers serAF/serAR, serBF/serBR and serCF/serCR, respectively. Primers pTrc101R/pTrc101F were used to amplify the backbone of the plasmid pTrc101. The *serABC* genes were cloned into pTrc101 by circular polymerase extension cloning (CPEC) [33], resulting in plasmid pTSer. The *pgk* gene was amplified with the primers pgkF/pgkR from *E. coli* genome and ligated into the pTSer and pTrc101, creating pTPSer and pTpgk. *E.coli* strains were transformed with the plasmids for PHB production or activate the SD pathway, for example, JM109 (pBHR68) represent the JM109 haboring the pBHR68 plasmid.

**Table 4:** **Strains and plasmids used in this study**

**Strain or plasmid**	**Relevant genotype** ^**b**^	**Source or reference**
Strains		
*E. coli* DH5α	*Coli* Genetic Stock Center strain (CGSC) No. 12384	CGSC^a^
*E. coli* JM109	*recA1*, *endA1*, *gyrA96*, *thi*, *hsdR17*, *supE44*, relA1, Δ(*lac proAB*)/*F’* [*traD36*, *proAB* ^*+*^, *lac* ^*q*^ *lacZ*ΔM15]	TaKaRa (Dalian, China)
SD01	SD01, P_*sdaA*_::P*trc*	This study
SD02	SD02, SerC:: P*trc*-*pgk*-*SerABC*	This study
SD03	SD02, P_*edd*_::P_*J23119*_	This study
SD04	SD02, P_*edd-eda*_::P_*J23119*_, P_*zwf*_::P_Trc-162_	
SD06	SD02, P_*aceE*_::P_Trc-162_	This study
SD07	SD04, P_*aceE*_::P_Trc-162_	This study
Tet-Trc-162	JM109, *tetA*-*trc*-M1-162-*glyA*	Lab collection
Plasmids		
pTrc101	Expression vector, pSC101 replication, constitutive *trc* promoter, Cm^r^	This study
pTSer	pSC101 replication, Cm^r^, P*trc* -*serA*-*serB*-*serC*	This study
pTPSer	pSC101 replication, Cm^r^, P_*trc*_- *pgk*-*serA*-*serB*-*serC*	This study
pTPgk	pSC101 replication, Cm^r^, P_trc_-*pgk*	This study
pBHR68	pBluscript SK(-) derivative, *phbA*_*Re*_ , *phbB*_*Re*_, *phbC*_*Re*_ cloned from *R. eutropha*	[34]
pTKRED	pSC101 replication, temperature sensitive replication origin, *Spc* ^*r*^, P_*araBAD*_-driven I-SceI gene, Red recombinase expression plasmid, lac-inducible expression	[35]
pTKS/CS	p15A replication, *Cm* ^*r*^, *Tet* ^*r*^, I-SceI restriction sites	[35]

^a^Coli Genetic Stock Center.

^b^ *Abbreviations:* *Amp* ampicillin, *Cm* chloramphenicol, *Tet* tetracycline, *Spc* spectinomycin, *r* resistance.

### Genome replacement manipulation

**Figure 5:**
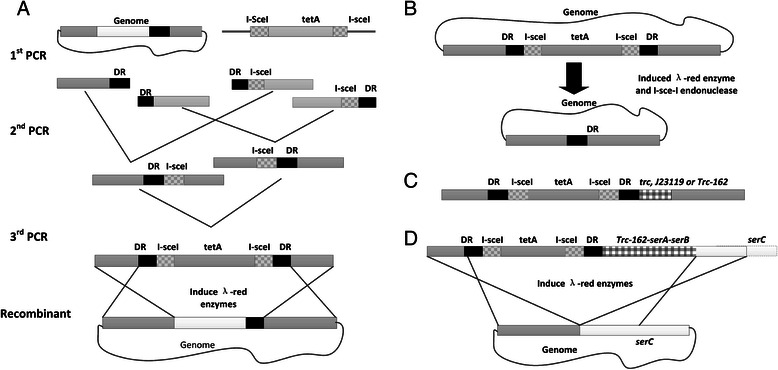
**Strategies for chromosomal replacement. A**. Genome editing cassettes are constructed by three rounds of PCR and recombinants after the first round of recombineering were selected by tetracycline; **B**. In the second step, the *tetA* marker was eliminated by simultaneous induction of I-SceI and Red recombinase expression. **C**. Fragments for promoter replacement or insertion; **D**. Fragments for P_trc-162_-*pgk-serABC* operon insertion. DR for duplicate region; I-sceI for I-SceI endonuclease recognition site.

The DNA fragment insertion or replacement strains were constructed by using the method reported by Lin *et al* with appropriate modifications [36]. The strategies of fragment construction were outlined in Figure 5. The final fragments were transformed into the competent cells with expression of the λ red recombination enzymes. The tetracycline resistant mutants were screened and confirmed by colony PCR. To induce I-SceI endonuclease expression and remove the resistance gene *tet*A from the genome, the positive colony was inoculated into 5 ml of LB medium with 100 μg/mL spectinomycin, 2 mM isopropyl-β-D-thiogalactopyranoside (IPTG), and 0.2% w/v L–arabinose. After overnight cultivation, cultures were diluted to appropriate concentration and plated on LB agar plates. The loss of *tetA* was confirmed by colony PCR. The technological process in detail was displayed in Figure 5. Primers used were listed in Table 5.

**Table 5:** **Primer sequence used in this study**

**Primer name**	**Primer sequence (5’→3’)**
serAF	TCTAGAGTCGAGCACAGCCGTATTCTAAGGAGGTCAAATGTGAGCCAGAATGGCCGTCC
serAR	GTAGTTTTGTCTCCGTTTAATTAGTCTTTAAGATCTTTAGCGAGCCAGATCCATCCACA
serBF	CGCTAAAGATCTTAAAGACTAATTAAACGGAGACAAAACTACATGCCTAACATTACCTG
serBR	CCTCCTTTATAAAATAGATTACTACCTTGGATCCTTACTTCTGATTCAGGCTGCCTGAG
serCF	GTAAGGATCCAAGGTAGTAATCTATTTTATAAAGGAGGAGGTAATGGCTCAAATCTTCA
serCR	CTTGCATGCCTGCAGGTCGACTTAACCGTGACGGCGTTCG
pTrc101R	TCTAGAGGATCCCCGGGTACCGAGCTC
pTrc101F	TCATGGTTGAGTTCGAACGCCGTCACGGTTAAGTCGACCTGCAGGCATGCAA
pgk_F	TCGAGCTCCGCACCACAAAAACGACACCATAGGGGGGCAAACGATGTCTGTAATTAAGA
pgk_R	GTGCGGAGCTCTTACTTCTTAGCGCGCTCTT
Primers for genetic manipulation	
SdaAp U_F	TATACCGCCTTCCGCCGTTG
SdaAp U_R	AATGAGCCGGATGATTAATTGTCAACAGCTCATTAGTCAGGGTTTCACACCAA
SdaAp T_F	ATGAGCTGTTGACAATTAATCATCCGGCTCATTACCCTGTTATCCCTACTAAGC
SdaAp T_R	TACTCCGTCGACTGTGTGACCACACATTATACGAGCCGGATGATTAATTGTCAACAGCTCATTAGGGATAACAGGGTAATGTACC
SdaAp L_F	ATAATGTGTGGTCACACAGTCGACGGAGTAACGACTCTCGTAAATAAGGAGTTTAAAGTGATTAGTCTATTCGACAT
SdaAp L_R	AAACCGGGAATACTGTCGAT
Tet U	AGCTGAGTCAGGAGATGCGG
Tet R	AGCTGTTTCCTGGTTTAAAC
Ser U_F	CTGGTCGAAACTCAATAACTCC
Ser U_R	TTAATTGTCAACAGCTCATCCGCATCTCCTGACTCAGCTTTCCCCTCACCACGTTGCGT
Ser L_F	GTACAGTACTTCAATTTGTTTAAACCAGGAAACAGCTATGTCTGTAATTAAGATGACCG
Ser L_R	AGCATTATCAGAGAGTTGCCAT
PaceEF U_F	GCCAGAACTTCGAATTGCTC
PaceEF U_R	TGTCAACAGCTCATCCGCATCTCCTGACTCAGCTGGGTTATTCCTTATCTATC
PaceEF L_F	GTACAGTACTTCAATTTGTTTAAACCAGGAAACAGCTATGTCAGAACGTTTCCCAA
PaceEF L_R	CCAGTTCCAGATTACCCGGAT
Pzwf U_F	TCCGCACTGAAAGAAATCGAA
Pzwf U_R	TGTCAACAGCTCATCCGCATCTCCTGACTCAGCTCGCATTCTCCTTAAGTTAAC
Pzwf L_F	GTACAGTACTTCAATTTGTTTAAACCAGGAAACAGCTATGGCGGTAACGCAAACAG
Pzwf L_R	ATTCAGTTTTGCCTCGCCAAG
Pedd U_F	CAGAAACCTTTAATCAGACGCATC
Pedd U_R	ACAGGGTAATCTAGGACTGAGCTAGCTGTCAACCATAAAGGATAAGCGCAGAT
Pedd T_F	CTTTATGGTTGACAGCTAGCTCAGTCCTAGATTACCCTGTTATCCCTACTAAGCACT
Pedd T_R	TATACCTAGGACTGAGCTAGCTGTCAACCATAAAGAAGGGATAACAGGGTAATGTACCA
Pedd L_F	ACAGCTAGCTCAGTCCTAGGTATAATGCTAGCACGAACAGGCGTTTCAGTCATAAATCC
Pedd L_R	CGCAACATGCTTTTCAAAGAGG
Primers for RT-PCR	
RTsdaA_F/R	CTATGAAGGCAGGTAAACAG/CGAGTAACGCTATCCAGTA
RTpgk_F/R	GTTCTAAAGTATCTACCAAACTG/ATACCACCACCAACAATC
RTSerA_F/R	CGATGGTGAG TGGAAACGCT/CGTAGGCAAGGATCTCATCA
RTSerB_F/R	GAGATCATGGACGGTAAA/ CAGAGTTTTCGCTTTGTA
RTSerC_F/R	TATTCCATCCTCAACGATA/GACCAGACCAGATAGATA
RTzwf_F/R	TTGCTAACTCCCTGTTTGT/CTTCTTCTGCCACGGTAA
RTaceE_F/R	TTACGAAGTTGCTGTCAT/AGCGTAGTGATGTAGTAGTA
RTaceF_F/R	AAATCCTGGTGAAAGTTG/TTCCATAGAAGCCTTGTC
RTlpd_F/R	CAGCAAGAAATTCAACCT/CCTTCCATCGTCACATAA
RTrrsA_F/R	TACGACCAGGGCTACACACG/ATCCGGACTACGACGCACTT

For swapping the promoter of *zwf*, PDH complex operon and *pgk*-*serAB* fragment insertion, the *tetA* fragment was amplified from strain Tet-Trc-162, and fused with the up and low homologous flanks (Figure 5C). The low flank of *pgk*-*serABC* fragment was amplified from the plasmid pTPSer (Figure 5D). Fragment Trc-162 consisted of the *trc* promoter core sequence and M1-162[37] in tandem.

### Cultivation conditions

During strains and plasmids construction, cultures were grown at 30°C or 37°C, in Luria broth (per liter: 10 g tryptone, 5 g yeast extract, and 10 g NaCl) with or without agar (2%) as indicated. Minimal sodium medium (MS medium) with 1 g/L yeast extract was used as seed culture and shake flask medium which contained (in grams per liter): glucose/xylose/glycerol 20.0, (NH_4_)_2_SO_4_ 2.0, MgSO_4_ · 7H_2_O 0.4, Na_2_HPO_4_ 3.83, KH_2_PO_4_ 1.5, Fe(III)-NH_4_-citrate 0.05, CaCl_2_ 0.02, and 1 mL/L trace element [38]. When necessary, a final concentration of 10 μg/mL chloromycetin and/or 100 μg/mL ampicillin were added. Colonies were inoculated into 5-ml LB culture medium and grown at 37°C with shaking overnight. Then the culture was inoculated with 1% into 250-ml flask with 50 ml culture medium as seed culture grown at 37°C in MS medium for 12 h at 220 rpm on a rotary shaker. Seed culture was then inoculated into 500-mL flask with 100 ml culture medium (with the initial OD_600_ of 0.04) and grown at 37°C and 220 rpm on a rotary shaker for 48 h. Three biological replicates were performed to detect the accumulation of PHB. To assess the significant difference of the PHB accumulation, data was subject to Student’s t test analysis with *p* < 0.05 being significantly different.

### Analytical techniques

The growth of cell was monitored by measuring the OD_600_ with an ultraviolet spectrophotometer (Beijing Puxi Universal Co Ltd). Glucose in the fermentation broth was determined utilizing a SBA sensor machine (Institute of Microbiology, Shangdong, China). Bacteria were harvested by centrifugation at 8,000 × g for 10 min and then washed with distilled water. Cell dry weight (CDW) was measured after lyophilization and vacuum drying. PHB content was analyzed by gas chromatography (Persee, China) with an Agilent J&W Capillary GC column after methanolysis of lyophilized cells in chloroform. To determine the concentration of pyruvate, acetate, glycerol and xylose, culture samples were centrifuged at 12,000 × g for 5 min and the aqueous supernatant used for HPLC analysis on an Agilent 1100 Series HPLC system equipped with an Aminex HPX-87H anion exchange column (Bio-Rad Laboratories, Richmond, CA, USA) and refractive index detector. A mobile phase of 5 mM H_2_SO_4_ at a 0.4 mL/min flow rate was used.

For the determination of intracellular acetyl-CoA, 40 mL mid-exponential phase cell culture was taken into precooled centrifuge tubes and centrifuged at 8000 g and 4°C for 10 min. The cell pellets were washed with 40 mL 100 mM Tris-HCl buffer (pH 8.0). Acetyl-CoA was analyzed by HPLC as previous reported [39,40]. For determination of intracellular NADP^+^ and NADPH, 10 mL mid-exponential phase cell culture was taken into precooled centrifuge tubes and centrifuged at 8000 g and 4°C for 10 min. The intracellular NADP^+^ and NADPH were analyzed by HPLC as previous reported [41].

### Quantitative real-time reverse transcription (RT)-PCR analysis

The recombinant strains harboring pBHR68 plasmid were cultured with the same fermentation media and culture condition with 1% (w/v) glucose. Cells were harvested when OD_600_ reached 1. Total mRNA were extracted using the RNAprep pure Cell/Bacteria Kit (Tiangen, Beijing, China) as described by the manufacturer. The cDNA was amplified using FastQuant RT Kit (Tiangen, Beijing, China) with the total mRNA as the templates. Samples were then analyzed using a Light Cycler® 480 II (Roche, Basel, Switzerland) with RealMasterMix (SYBR Green I) (Tiangen, Beijing, China). Quantity real-time PCR (qPCR) amplification primers were designed and were listed in Table 5. The *rrsA* gene was selected as internal standard for normalization and three biological replicates were performed. The obtained data were analyzed by using the 2^-ΔΔCt^ method described previously [42].
